# Genomic Diversity and Hotspot Mutations in 30,983 SARS-CoV-2 Genomes: Moving Toward a Universal Vaccine for the “Confined Virus”?

**DOI:** 10.3390/pathogens9100829

**Published:** 2020-10-10

**Authors:** Tarek Alouane, Meriem Laamarti, Abdelomunim Essabbar, Mohammed Hakmi, El Mehdi Bouricha, M. W. Chemao-Elfihri, Souad Kartti, Nasma Boumajdi, Houda Bendani, Rokia Laamarti, Fatima Ghrifi, Loubna Allam, Tarik Aanniz, Mouna Ouadghiri, Naima El Hafidi, Rachid El Jaoudi, Houda Benrahma, Jalil El Attar, Rachid Mentag, Laila Sbabou, Chakib Nejjari, Saaid Amzazi, Lahcen Belyamani, Azeddine Ibrahimi

**Affiliations:** 1 Medical Biotechnology Laboratory (MedBiotech), Bioinova Research Center, Rabat Medical and Pharmacy School, Mohammed Vth University, Rabat 10100, Morocco; m_laamarti@yahoo.com (M.L.); abdelmounim.essabbar@um5s.net.ma (A.E.); gml.hakmi@gmail.com (M.H.); elmehdi.bouricha@gmail.com (E.M.B.); walidchemao@gmail.com (M.W.C.-E.); souad.kartti@um5s.net.ma (S.K.); nassma.boumajdi@gmail.com (N.B.); hbendani.houda@gmail.com (H.B.); fatima.ghrifi@um5s.net.ma (F.G.); louabna.allam@um5s.net.ma (L.A.); t.aanniz@um5s.net.ma (T.A.); mouna.ouadghiri@um5.ac.ma (M.O.); n.elhafidi@um5s.net.ma (N.E.H.); r.eljaoudi@um5s.net.ma (R.E.J.); 2 Medical Biotechnology Center, Moroccan Foundation for Science, Innovation & Research (MAScIR), Rabat 10100, Morocco; r.laamarti@mascir.ma; 3 Faculty of Medicine, Mohammed VI University of Health Sciences (UM6SS), Casablanca 82403, Morocco; hbenrahma@um6ss.ma; 4 Riad Laboratory, City Center Hay Riad, Rabat 10112, Morocco; laboratoireriad@gmail.com; 5 Biotechnology Unit, Regional Center of Agricultural Research of Rabat, National Institute of Agricultural Research, Rabat 10101, Morocco; rachid.mentag@inra.ma; 6 Microbiology and Molecular Biology Team, Center of Plant and Microbial Biotechnology, Biodiversity and Environment, Faculty of Sciences, Mohammed V University, Rabat 10000, Morocco; l.sbabou@um5s.net.ma; 7 International School of Public Health, Mohammed VI University of Health Sciences (UM6SS), Casablanca 82403, Morocco; cnejjari@um6ss.ma; 8 Laboratory of Human Pathologies Biology, Faculty of Sciences, Mohammed V University, Rabat 10000, Morocco; amzazi@gmail.com; 9 Emergency Department, Military Hospital Mohammed V, Rabat Medical and Pharmacy School, Mohammed Vth University, Rabat 10112, Morocco; l.belyamani@um5s.net.ma

**Keywords:** COVID-19, SARS-CoV-2, genomic diversity, divergence, hotspot mutations, spike protein, vaccine

## Abstract

The COVID-19 pandemic has been ongoing since its onset in late November 2019 in Wuhan, China. Understanding and monitoring the genetic evolution of the virus, its geographical characteristics, and its stability are particularly important for controlling the spread of the disease and especially for the development of a universal vaccine covering all circulating strains. From this perspective, we analyzed 30,983 complete SARS-CoV-2 genomes from 79 countries located in the six continents and collected from 24 December 2019, to 13 May 2020, according to the GISAID database. Our analysis revealed the presence of 3206 variant sites, with a uniform distribution of mutation types in different geographic areas. Remarkably, a low frequency of recurrent mutations has been observed; only 169 mutations (5.27%) had a prevalence greater than 1% of genomes. Nevertheless, fourteen non-synonymous hotspot mutations (>10%) have been identified at different locations along the viral genome; eight in ORF1ab polyprotein (in nsp2, nsp3, transmembrane domain, RdRp, helicase, exonuclease, and endoribonuclease), three in nucleocapsid protein, and one in each of three proteins: Spike, ORF3a, and ORF8. Moreover, 36 non-synonymous mutations were identified in the receptor-binding domain (RBD) of the spike protein with a low prevalence (<1%) across all genomes, of which only four could potentially enhance the binding of the SARS-CoV-2 spike protein to the human ACE2 receptor. These results along with intra-genomic divergence of SARS-CoV-2 could indicate that unlike the influenza virus or HIV viruses, SARS-CoV-2 has a low mutation rate which makes the development of an effective global vaccine very likely.

## 1. Introduction

The year 2019 ended with the appearance of groups of patients with pneumonia of unknown cause. Initial evidence suggested that the outbreak was associated with a seafood market in Wuhan, China, as reported by local health authorities [1]. The results of the investigations led to the identification of a new coronavirus in affected patients [2]. Following its identification on the 7 January 2020 by the Chinese Center for Disease Control and Prevention (CCDC), the new virus and the disease were officially named SARS-CoV-2 (for severe acute respiratory syndrome coronavirus-2) and COVID-19 (for coronavirus disease 19), respectively, by the World Health Organization (WHO) [3]. On 11 March 2020, WHO publicly announced the SARS-CoV-2 epidemic as a global pandemic.

This virus is likely to remain and continue to spread unless an effective vaccine is developed, or a high percentage of the population is infected in order to achieve collective immunity. The development of a vaccine is a long process and is not guaranteed for all infectious diseases. Indeed, some viruses such as influenza and HIV have a high rate of genetic mutations, which makes them prone to antigenic leakage [4,5]. It is therefore important to assess the genetic evolution of the virus and more specifically the regions responsible for its interaction and replication within the host cell. Thus, identifying the conserved and variable regions of the virus could help guide the design and development of anti-SARS-CoV-2 vaccines.

SARS-CoV-2 is a single-stranded positive-sense RNA virus belonging to the genus *Betacoronavirus*. The genome size of SARS-CoV-2 is approximately 30 kb and its genomic structure has followed the characteristics of known genes of the coronavirus [6]. The ORF1ab polyprotein is covering two-thirds of the viral genome and cleaved into many nonstructural proteins (nsp1 to nsp16). The third part of the SARS-CoV-2 genome codes for the main structural proteins; spike (S), envelope (E), nucleocapsid (N), and membrane (M). In addition, six ORFs, namely ORF3a, ORF6, ORF7a, ORF7b, ORF8, and ORF10, are predicted as hypothetical proteins with no known function [7].

Protein S is the basis of most candidate vaccines; it binds to membrane receptors in host cells via its RBD and ensures a viral fusion with the host cells [8]. Its main receptor is the angiotensin-converting enzyme 2 (ACE2), although another route via CD147 has also been described [9,10]. The glycans attached to S protein assist the initial attachment of the virus to the host cells and act as a coat that helps the virus to evade the host’s immune system. In fact, a previous study has shown that glycans cover about 40% of the surface of the spike protein. However, the ACE2-RBD was found to be the largest and most accessible epitope [11]. Thus, it may be possible to develop a vaccine that targets the spike receptor-binding domain (RBD), provided it remains accessible and stable over time; hence, the importance of monitoring the introduction of any mutation that could compromise the potential effectiveness of a candidate vaccine.

This study aims to deepen our understanding of the intra-genomic diversity of SARS-CoV-2, by analyzing the mutational frequency and divergence rate in 30,983 genomes from six geographic areas (Africa, Asia, Europe, North and South America, and Oceania), collected during the first five months after the onset of the virus. These analyses generate new datasets providing a repository of genetic variants from different geographic areas, with particular emphasis on recurrent mutations and their distribution along the viral genome as well as estimating the rate of intraspecific divergence while evaluating the adaptation of SARS-CoV-2 to its host and the possibility of developing a universal vaccine.

## 2. Results

### 2.1. Diversity of Genetic Variants of SARS-CoV-2 in Different Geographic Areas

**Figure 1:**
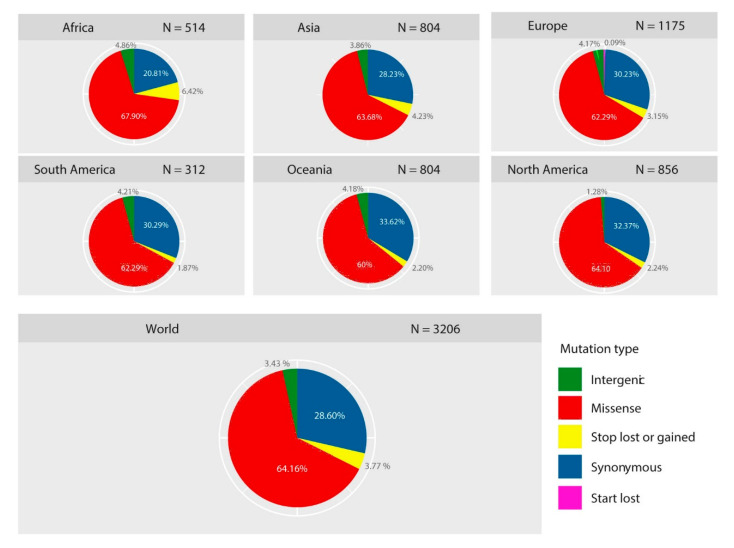
Prevalence and distribution of types of mutations in different geographic regions. Pie charts showing the global and continent-stratified distribution of the mutation types identified in the 30,983 SARS-CoV-2 genomes. The prevalence of each type of mutation is uniform across the six geographic areas and missense mutations were the most frequent type. Color codes represent the type of mutations.

A total of 30,983 SARS-CoV2 genomes from 79 countries in six geographic areas (Africa, Asia, Europe, North and South America, and Oceania) were included in this analysis. According to the GISAID database, the date of collection of the strains was within the first five months following the onset of SARS-CoV-2 (Supplementary Table S1). A total of 3206 variant sites were detected compared to the reference genome Wuhan-Hu-1/2019 (Supplementary Table S2). Then, we analyzed the type of each mutation, highlighting the prevalence of these mutations both in all genomes (worldwide) and in each of the geographic areas studied (Figure 1). Worldwide, 67.96% of mutations had a non-synonymous effect (64.16% have missense effects, 3.77% produce a gain or loss of stop codon, and 0.33% produce a loss of start codon), 28.60% were synonymous, while 3.43% of the mutations were localized in the intergenic regions, mainly in the untranslated regions (UTR). Likewise, the comparison between the six geographic areas shows a similar trend with a uniform distribution of the prevalence of each type of mutation.

**Figure 2:**
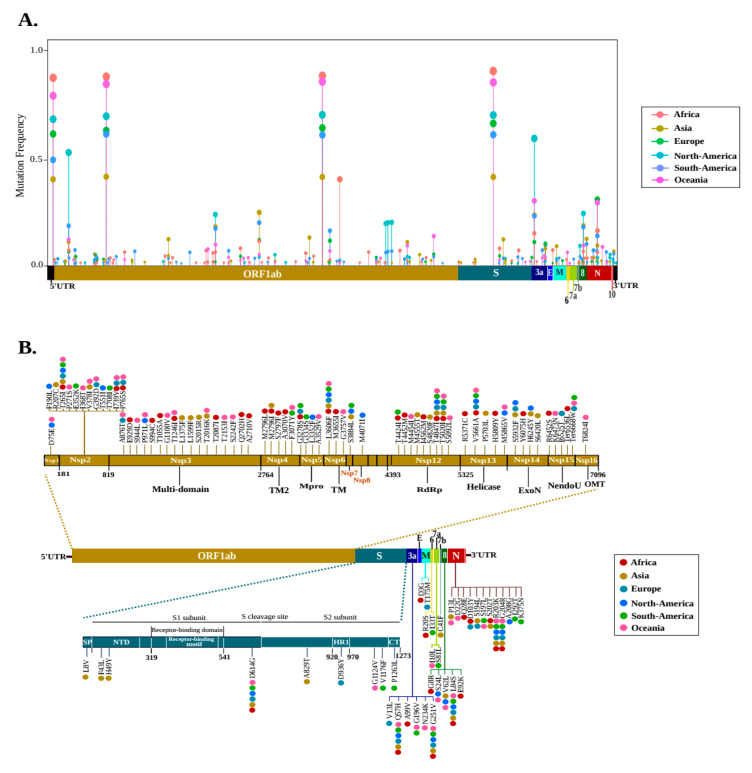
Distribution of recurrent mutations across the SARS-CoV-2 genome. (**A**) Lollipop plot illustrating the location of mutations with a frequency greater than 0.01 of the total genomes of each geographic area. All types of mutations are included (non-synonymous, synonymous, and intergenic). The mutation frequency was estimated for each of them, by normalizing the number of genomes harbored in a given mutation in a geographic area, per the total number of genomes recovered by geographic area. (**B**) Schematic representation illustrating the distribution of non-synonymous mutations (with a frequency >0.01) along the viral genome. Amino acid mutations are shown by vertical lines. Colored dots represent geographic areas.

The frequency of mutations in the six geographic areas was estimated by normalizing the number of genomes carrying a given mutation per the total number of genomes recovered by geographic area. Only 169 (5.27%) variant sites were found with a frequency greater than 0.01 (Figure 2A, Supplementary Table S3), and were distributed in six geographic areas as follows: 69 in Oceania, 65 in Africa, 54 in Asia, 31 in Europe, 43 in North America, and 43 in South America. Focusing on non-synonymous mutations (with a frequency >0.01), 3.34% (*n* = 107) of the total mutations were identified (Figure 2B).

The polyprotein ORF1ab contained approximately two thirds of these mutations (63.55%; *n* = 68) and distributed in thirteen non-structural proteins; nsp3-Multi-domain: 15.89%, nsp2: 11.21%, nsp12-RNA-dependent RNA polymerase (RdRp): 8.41%, nsp4-transmembrane domain-2 (TM-2): 4.67%, nsp13-helicase: 4.67%, nsp15-endoribonuclease (NendoU): 4.67%, nsp5-main proteinase (Mpro): 3.74%, nsp14-exonuclease (ExoN): 3.74%, nsp6-TM: 2.80%, nsp1: 0.93%, nsp7: 0.93%, nsp8: 0.93%, and nsp16-2’-O-ribose methyltransferase (OMT): 0.93%. The rest (36.45%) were distributed in eight proteins, including N (11.21%), S (8.41%), ORF3a (5.61%), ORF8 (4.67%), M (1.87%), ORF6 (1.87%), ORF7a (1.87%), and ORF7b (0.93%).

Comparative analysis of these non-synonymous mutations shows only nine that have been shared in the six geographic areas: T265I (nsp2), L3606F (in nsp6-TM) T4847I (in nsp12-RdRp), D614G (in S), R203K-G204R (in N), Q57H-G251V (in ORF3a), and L84S (in ORF8).

It is also interesting to note that none of the nine non-synonymous mutations (>0.01) of S protein was localized in RBD. The 36 non-synonymous mutations (35 with a missense effect and 1 with a stop gain effect) found in this area had a low frequency (<0.01) across all genomes (Supplementary Table S4). Among them, only two mutations were shared between genomes of different geographic areas; the V367F mutation was identified in Europe, Asia, and North America, the V367F mutation has been identified in Europe, Asia, and North America, while P491L in Asia and Oceania.

### 2.2. Geographical Distribution of the SARS-CoV-2 Hotspot Mutations

**Figure 3:**
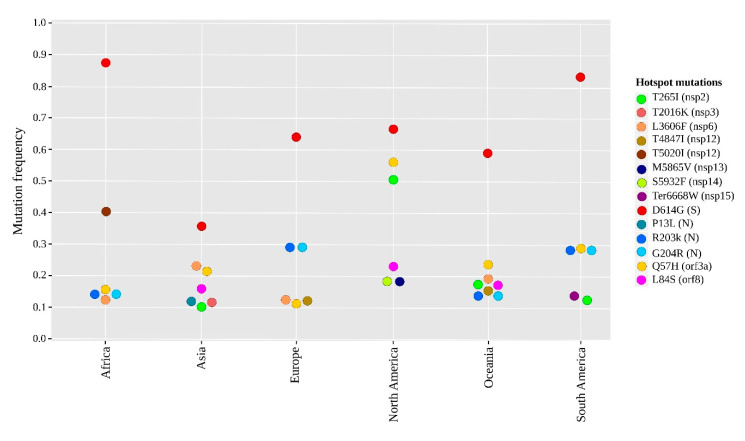
Frequencies of recurrent hotspot mutations per geographic area. Distribution of fourteen non-synonymous mutations with a frequency >0.1 of the genomes subdivided into six geographical areas; Africa (*n* = 6), Asia (*n* = 7), Europe (*n* = 6), North America (*n* = 6), Oceania (*n* = 8), South America (*n* = 6). The locations of mutations in viral proteins with their color codes are indicated in the legend.

Comparative genomic analysis of each geographic area revealed fourteen non-synonymous mutations with a frequency greater than 0.1 and considered as hotspot mutations (Figure 3). Eight mutations of them were found in the ORF1ab polyprotein, distributed in seven regions coding for nsp2 (T265I), nsp3-Multi-domain (T2016K), nsp6-TM (L3606F), nsp12-RdRp (T5020I and T4847I), nsp13-helicase (M5865V), nsp14-ExoN (D5932T) and nsp15-NendoU (Ter6668W). Moreover, three mutations in N protein (R203K, G204R, and P13L) and one in each of the three proteins; S (D614G), ORF3a (Q57H), and ORF8 (L84S).

Different patterns of these non-synonymous hotspot mutations were observed between the six geographic regions. Only two mutations were common in the six geographical regions: The high-frequency mutation D614G (in S) and the Q57H mutation (in ORF3a). Seven mutations were more frequent in a single geographic region, including two mutations T2016K (in nsp3-Multi-domain) and P13L (in N) in Asia, two mutations M5865V (in nsp13-helicase) and D5932T (in nsp14-ExoN) in North America, one T5020I (in nsp12-RdRp) in Africa, one T4847I (in nsp12-RdRp) in Europe, and one Ter6668W (in nsp15-NendoU) in South America. However, the other five non-synonymous hotspot mutations were variable between the six geographical regions, including two R203K and G204R (in N) that were particularly predominant in Africa, Europe, South America, and Oceania; whereas, L3606F (in nsp6-TM) was common in Africa, Asia, Europe, and Oceania. Thus, L84S (in ORF8) was found in Asia, North America, and Oceania. In addition, T265I (in nsp2) was frequent in Asia, North America, South America, and Oceania.

### 2.3. The Distribution of Hotspot Mutation Patterns of SARS-CoV-2 over Time

**Figure 4:**
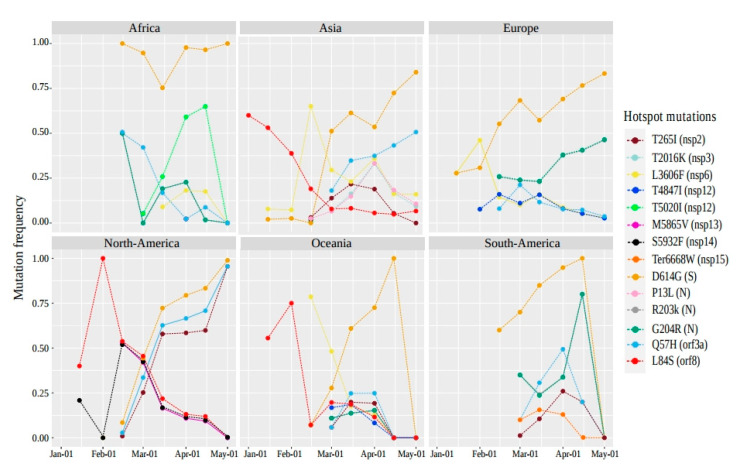
Tracking hotspot mutations over time per geographic area. Hotspot mutation frequencies were plotted for each of them over a period of 15 days in each geographic area, first by normalizing the number of genomes harboring a given mutation in a period of 15 days, per the total number genomes recovered at this time for each of six continents. The X axis represents the time measured in 15 days and the Y axis represents the frequencies of the genomes harboring the hotspot mutations.

A different pattern of hotspot mutations over time is clearly distinguished between the six continents from January to May 2020 (Figure 4). The number of mutations was normalized for each of the six geographic areas for 15 days per the total number of genomes recovered during this period (depending on the date of sample collection provided by GISAID).

The L84S mutation (red) was the first mutation observed (early January in Asia) and the most propagated between January–February in North America and Oceania, before starting to drop dramatically after. Remarkably, the D614G (orange) was the most common on six continents. This mutation first appeared on 24 January 2020, in Asia (China), after four days it was observed in Europe (Germany), and then gained its predominance over time, when the outbreak of positive cases was reported in the United States and Canada (Supplementary Table S2). The highest recorded frequency of D614G was in Africa; this mutation was present in most African genomes from late February to May, with a small fluctuation in frequency in mid-March. On the same continent, the frequency of genomes containing the T5020I (lawn green) mutation increased until the end of April before disappearing in May. The other three mutations (Q57H-sky blue, R203K-gray, and G203R-green) started with a high frequency (0.5) at the beginning of March and decreased slightly over time. While in Europe, except for D614G, the two R203K-G203R mutations were the most prevalent, showing continuous growth with the same frequencies over time. In addition, the L3606F (yellow) mutation showed an increase during February, followed by a decrease to nearly 0.1 frequency in early May.

For North America, three hotspot mutations, D614G, Q57H, and T265I (garnet red), continued to increase with the same trend after their appearance. Unlike the other three mutations (L84S, S5932F-black, and M5865V-dark pink), their frequencies were reduced over time especially from mid-February. Interestingly, a different pattern of hotspot mutations was observed in South America and Oceania between March and April. Focusing on South America, a new stop-loss Ter6668W (dark orange) hotspot mutation (in nsp15-NendoU) was reported in North American genomes from March and decreased one month later, while at that date, the frequency of the co-occurring mutations R203K-G203R was increased over time. Overall, the fourteen hotspot mutations were seen between January–March and most of them gained their frequency outside of Asia.

### 2.4. Mutagenesis of D614G and Impact of RBD Mutations on the Binding Ability of Spike to ACE2

**Figure 5:**
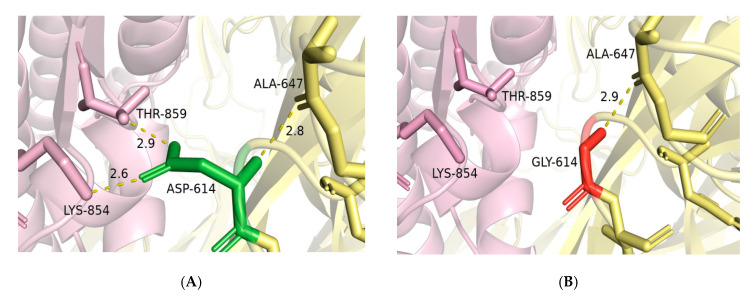
Comparison of spike wild-type residue ASP-614 (**A**) and the mutated GLY-614 (**B**). ASP-614 (green sticks) in subunit S1 (yellow) is involved in two hydrogen bonds with THR-859 and LYS-854 from the S2 subunit (pink). The substitution of ASP by GLY at position 614 causes the loss of the two hydrogen bonds between S1 subunit and THR-859 and LYS-854 in the S2 subunit (pink).

As shown the Figure 5, the non-synonymous D614G mutation did not have an impact on the two- or three-dimensional structure of the spike glycoprotein. However, D614 residue in the wild-type spike is involved in three hydrogen bonds; one with A647 in the same subunit (S1) and two bonds with THR-859 and LYS-854 located at S2 subunit of the adjacent protomer (Figure 5A). The substitution of D614 by G in the mutant spike resulted in the loss of the two hydrogen bonds with THR-859 and LYS-854 in the S2 subunit of the adjacent protomer (Figure 5B). Such modification could result in a weak interaction between S1 and S2 subunits and thus increase the rate of S1/S2 cleavage, which would improve the virus entry to host cells.

To evaluate the effect of RBD mutations on the binding affinity of the spike protein to ACE2, the Molecular Mechanics-Generalized Born Surface Area (MM-GBSA) method was employed to calculate the binding affinity of 35 spike mutants to ACE2 (except for the stop-gain mutation). Four mutations potentially enhanced the binding affinity of spike/ACE2 complex by a binding affinity change (ΔΔG) < −1.0 kcal/mol, while nine were shown to potentially reduce its affinity by a ΔΔG > 1.0 kcal/mol (Table 1). However, the remaining 22 did not significantly affect the binding affinity of spike to ACE2.

### 2.5. Clustering and Divergence of SARS-CoV-2 Genomes

To compare the mutational profile similarity between the 79 countries, we used the Jaccard distance as a suitable metric for clustering, allowing the overall similarity measure, ranging from 0 (identical) to 1 (no overlap). We first calculated the mutational frequency in each country individually, then the Jaccard method was used to measure the distances between countries (see Materials and Methods). Figure S1 shows the pairwise similarity between countries, scaled from 0 (light yellow) to 1 (red). The clustering between the 79 countries showed two main clusters, each subdivided into several sub-clusters (SCs), and these two clusters included countries of the six continents. In addition, the countries in cluster 2 were closer to each other than those in cluster 1, demonstrating high genetic similarities between strains from these countries. We also observed fifteen SCs with a distance of less than 0.5, which corresponds to at least a 50% overlap (Table 2), ten of which belonged to cluster 2. Remarkably, most of the countries grouped in these fifteen SCs were geographically close, of which eleven SCs included countries of the same continent, especially Asia and Europe. The results of clustering between the 79 countries (country by country) are detailed in Table S5.

**Figure 6:**
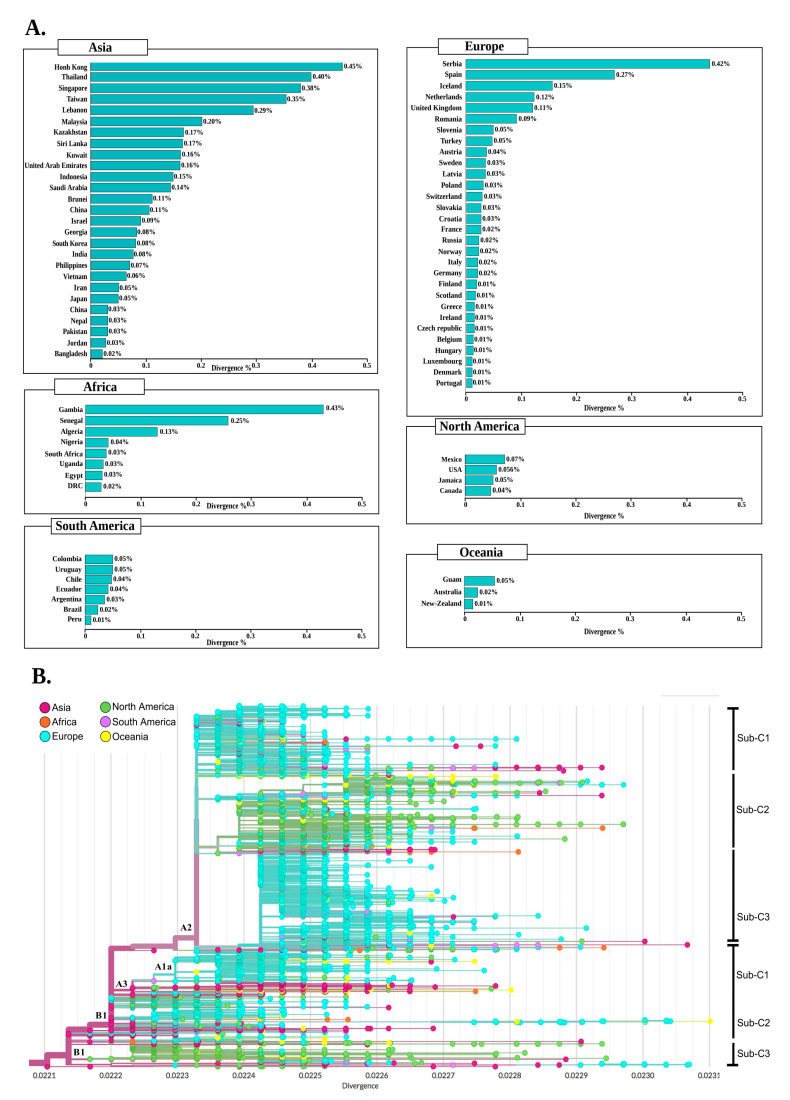
Divergence of SARS-CoV-2 genomes from different geographic areas compared with the genome reference Wuhan-Hu-1/2019. (**A**) The bar graph illustrating the divergence (measured in percentage) of the SARS-CoV-2 genomes of each country compared to the reference genome Wuhan-Hu-1/2019. The divergence calculation method is detailed in the Materials and Methods section. (**B**) The phylogenetic divergence tree of the 30,983 SARS-CoV-2 genomes grouped into six geographic regions. The length of the branches shows the divergence and the color codes indicate the six geographical areas.

Meanwhile, the intraspecific divergence of SARS-CoV-2 was also assessed in the genomes of each country compared to the genome reference Wuhan-Hu-1/2019. As shown in Figure 6A, the overall circulating strains in more than 50 countries seem to have a divergence percentage of less than 0.1%, which indicates that the majority of SARS-CoV-2 genomes have developed less than 18 mutations in them. The highest percentage of divergence in Asia, Europe, North America, South America, Oceania, and Africa was observed in Hong Kong (0.45%), Serbia (0.42%) Mexico (0.07%), Colombia (0.05%), Guam (0.05%), and Gambia (0.43%), respectively. While the lowest percentage was shown in Portugal (0.01), Canada (0.05%), Bangladesh (0.02%), Peru (0.01%), New Zealand (0.02%), and DRC (0.03%).

Moreover, the phylogenetic divergence tree (Figure 6B) shows that the highest rate was among genomes from Asia, followed by Europe, and North America.

In Asia, most strains showed a divergence of 0.0221 to 0.0231. Likewise, European strains clustered between 0.0223 and 0.0231, while North American strains had a divergence of 0.0221 to 0.0230. Using the Nextstrain clade nomenclature, we can identify two main clades with different divergence profiles; first and most divergent “A2” clade, although the first strain observed was from China. This clade mainly contained genomes from Europe. The second “B1” clade appeared to be less divergent and to a large extent included Asian strains. Nevertheless, the genomes of Africa, North America, and South America were scattered across the phylogenetic divergence tree without a specific coating.

The rate of divergence also varied within clades: A2 included three subclades, the sub-c2 harboring the Q57H mutation, with a divergence of 0.0224 to 0.0229, and mainly included strains from North America and Asia. The sub-c3 containing mostly European genomes shared the R203K mutation: In this subclade, a low rate of divergence was observed in all continents except Africa, while the greatest divergence was a strain from Taiwan (Asia) (>0.0223).

On the other hand, clade 2 (B1) harbored mainly genomes from North America and Asia, while the high divergence in this clade observed in Europe (France) with 0.0231. The sub-c2 and sub-c3 of this clade appeared to be the most diverse with the lowest divergence in the United Kingdom and the highest in Australia.

### 2.6. Phylogenetics and Spatio Dynamics of SARS-CoV-2

**Figure 7:**
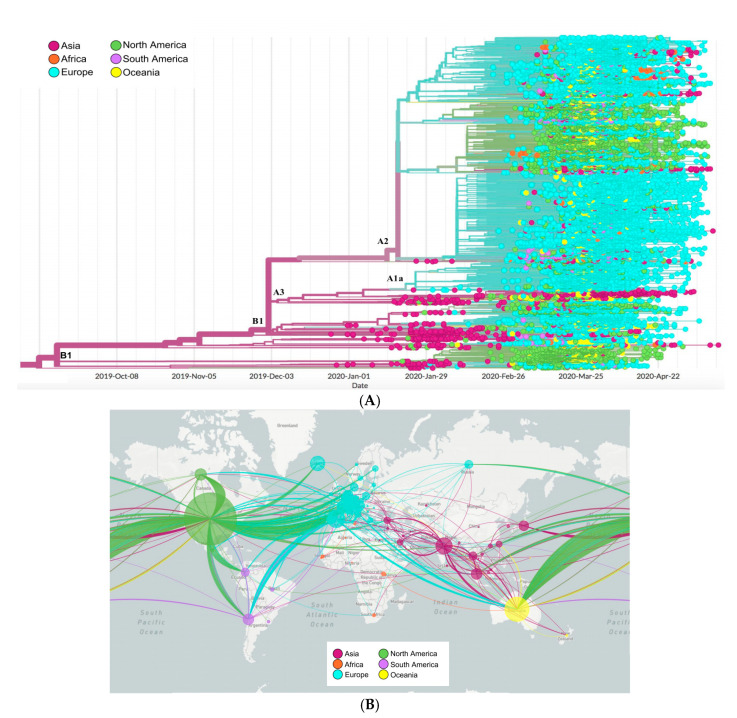
Phylogenetic tree and spatial dynamics of SARS-CoV-2. (**A**) Phylogenetic analysis of 30,983 SARS-CoV-2 genomes grouped into six geographic areas. The length of the branches represents the distance in time. (**B**) Phylodynamic analysis representing the propagation and evolution of 30,983 SARS-CoV-2 genomes in different geographic areas. The color codes represent the six geographic areas.

The topology of the maximum likelihood phylogenetic tree (Figure 7A) shows a clear clustering: one cluster containing mainly Asian strains, while the second containing European strains with a specific clade sharing the D614G mutation. For each cluster, we identified different clades: cluster 1 containing two main clades A1a and B1 harboring mainly strains from Asia, North America, and Asia, Europe, respectively. However, cluster 2 harbored three clades: B2, A2, A2a without a specific pattern.

The distribution of African genomes across the phylogenetic tree showed a close relationship with different continents. In the first clade, African genomes (mainly from South West Africa) clustered with Asia and showed the lowest divergence rate. Meanwhile, genomes clustering in the European clade shared the three-pattern mutations mostly common in Europe: G28881A, G28882A, and G28883C.

The map (Figure 7B) shows the spatio-dynamics of SARS-CoV-2 and provides an insight into the viral strain’s potential geographical origin based on the sample used and displays a complex and interconnected network of strains. From these samples, strains from Asia appear to have diverged and resulted in other strains in all the investigated regions. European strains seem to have given rise to those in North America, South America, and Africa, with multiple divergent strains within Europe itself. Similarly, with less frequency, strains from South and North America appear to be related to some divergent strains in Europe and Asia.

## 3. Discussion

Due to the rapid spread and mortality rate of the new SARS-CoV-2 pandemic, the development of an effective vaccine against this virus is of a high priority [12]. The availability of the first viral sequence derived during the COVID-19 epidemic, Wuhan-Hu-1, was published on 5 January 2020. From this date, numerous vaccination programs were launched [12,13]. Furthermore, drugs and vaccines should target relatively invariant and highly constrained regions of the SARS-CoV-2 genomes, to avoid drug resistance and vaccine escape [14]. For this, monitoring genomic changes in the virus are essential and play a pivotal role in all of the above efforts, due to the appearance of genetic variants, which could affect the efficacy of vaccines. In this study, we investigated the genetic diversity in 30,983 complete SARS-CoV-2 genomes isolated from 79 countries belonging to the six continents, while evaluating the possibility of developing an effective universal vaccine.

Our results showed three different situations of the identified mutations: (i) The mutations that have developed and are gaining a predominance in the six geographic areas; (ii) mutations which were predominant only in certain geographic regions; and (iii) mutations apparently expanding, but low in frequency in all isolates studied. From this third situation, it is interesting to note that a low rate of recurrent mutations was found across genomes, with only 5.27% of the total mutations have a frequency greater than 0.01, while 94.73% had a frequency of less than 0.01, of which 49.68% were single mutations (specific to a genome). In line with previous reports, our results show strong evidence that, so far, the evolution of SARS-CoV-2 has evolved in a non-deterministic process and that this diversification has mainly been due to random genetic drift which plays a dominant role in the spread of low-frequency mutations [15,16,17,18,19], suggesting that there was no strong selective pressure exerted on SARS-CoV-2 by the human population. Although the hotspot mutations are motivated by positive selection, which could indicate that the substitution of a specific amino acid offers an adaptive advantage under particular conditions [20]. Our study showed that more than half of the hotspot mutations identified in the SARS-Vo2 genomes gained their predominance outside of Asia; including the hotspot mutation Q57H (in ORF3a), until early March, which had not yet been observed among isolates from China, while it emerged before that date in Europe and also spread in isolates from North America. Likewise, seven other hotspot mutations with high frequency in different geographic areas (except Asia); including the double mutations R203K-G204R and double mutations of the N protein (in Europe, South America, and Oceania), M5865V of nsp13-helicase, and D5932T of nsp14-ExoN (in North America), and T5020I of nsp12-RdRp (in Africa). Hotspot mutations, due to their increased frequency in different geographic areas, are considered an important criterion for defining and characterizing emerging clades [21,22].

As a whole, a low rate of intra-genomic divergence of SARS-CoV-2 (<0.5%) was found between all the countries studied. Compared to different geographic areas, the high rate of divergence in Asian countries could be due to multiple sources of infection with different strains at the start of the epidemic. This could suggest an early introduction and rapid spread of genetically close variants to the original strain in continents with high infection rates, such as Europe and North America, which founded the virus’s first transmission networks [23,24]. Rapid transmission means a single source leading to multiple infections, thus giving the virus fewer life cycles to change: This is consistent with a previous study describing a continued tendency of the virus to diverge over time [25]. Furthermore, the dynamics of transmission showed that the least divergent African variants were grouped with Asian strains, while the most divergent were grouped with Europe and North America. This distribution points to different sources of infection [26,27]. South America’s genomes appear to originate from North America and Europe, showing a close clustering with Europe in low and high divergence strains, which is concordant with a previous study [28]. In contrast, certain strains from Oceania allow poor monitoring of the origin of the infection, but show a close relationship with the genomes of Europe. Overall, the North American and European genomes appear to be responsible for most of the spread of the disease. Besides the divergence, the intra-genomic clustering between the 79 countries did not have a clear pattern regarding their geographic distributions, reflecting the effect of migration and globalization as previously reported [29,30].

As the virus spreads more widely around the world, it is important to monitor and assess mutations that could be of potential concern as an early warning system to consider as vaccine studies progress. The S protein is a major target for vaccines and therapeutics, due to its key role in mediating virus entry and its immunogenicity trait [8,31]. Analysis of protein S revealed a high-frequency mutation (D614G) with a continuous trend over time in different geographic areas. This mutation is proximal to the S1 cleavage domain at position 614 which involved the change of a large amino acid residue (aspartic acid) to a small hydrophobic residue (glycine) and became widely dominant worldwide within a few months [32,33]. Our results showed that this mutation induces a loss of two hydrogen bonds between the S1 and S2 subunits of neighboring protomers and can, therefore, increase the rate of cleavage of these subunits in the pre-fusion state of spike protein to allow its conformational transition to the post-fusion state associated with membrane fusion upon virus entry [34,35]. Indeed, our structural modeling of this mutation has shown no substantial impact on the secondary or tertiary structure of the spike protein. Therefore, it is unlikely that G614D could affect the immunogenicity of RBD epitopes considered important in neutralizing antibodies [29,36]. Likewise, previous studies [29,30,37] reported that the antibodies generated from natural infection with mutant type D614 or G614 could carry out a neutralization cross, indicating that the locus is not critical for antibody-mediated immunity, so the D614G mutation is unlikely to have a major impact on the efficacy of vaccines in development, some of which exclusively target RBD region. To this end, the RBD of the spike protein allows the virus to bind to the ACE2 host receptor [38,39]. Mutations in this receptor are a likely pathway to evade antibody recognition, such as described in other viruses [40,41]. In all the genomes analyzed, 36 non-synonymous RBD mutations were identified and all these mutations had a low frequency (<0.01) in the genomes of six continents, which is consistent with several studies that have found that mutations are extremely rare in the RBD region [19,20,21,22,23,24,25,26,27,28,29,42,43]. The calculated binding-free energy of mutant RBDs of spike protein complexed with human ACE2 revealed only four RBD mutant types (D364Y, N440K, N450K, S477R) displaying a much lower binding-free energy (ΔG), indicating a significantly higher affinity for the ACE2, which could influence the pathogenicity of SARS-CoV-2. Of these four mutations, Ou et al. [44] previously reported that D364Y potentially enhances the binding of viral spike protein to ACE2, possibly due to the improved structural stabilization of the RBD beta-sheet scaffold.

Effective COVID-19 vaccines will be a permanent solution to viral infections, and it is likely that more than one strategy will be successful to this end [45]. RNA interference-based therapy (RNAi) could be an alternative in the fight against SARS-CoV-2 [46], where small interfering RNAs (siRNA, 20 to 25 nt in length) could affect the region highly conserved from SARS-CoV-2 and could also act as an inhibitor to suppress genetic disorders in the lungs [47]. The efficiency of siRNA to inhibit gene expression and replication by targeting the leader and spike coding sequence of SARS-CoV has already been demonstrated [48,49,50]. Alnylam Pharmaceuticals (USA) has designed and synthesized over 350 siRNAs targeting highly conserved regions of circulating SARS-CoV-2 genomes [51]. The main siRNA candidates will be evaluated for their antiviral activity in vitro and in vivo, leading to the selection of a candidate for development. It is interesting to note that the effects of siRNAs can be influenced by mutations. Chen et al. 2020 [52] reported nine potential target siRNA sequences in the SARS-CoV-2 genome. To this end, we analyzed the mutations present in these target sequences in the 30,983 genomes of our study. One to seven SNPs in each of the nine target sequences were found (Supplementary Table S6), hence the importance of monitoring the introduction of any mutations that could compromise the potential efficacy of siRNAs and candidate vaccines.

SARS-CoV-2 has only recently been discovered in the human population; adaptive processes could take years to occur. Although we cannot predict whether adaptive selection will be observed in this virus in the future, we can conclude that the currently circulating strains constitute a homogeneous viral population. We can therefore be cautiously optimistic that, so far, the genetic diversity of SARS-CoV-2 should not be an obstacle to the development of a universal vaccine candidate. Ongoing surveillance of SARS-CoV-2 genomic changes will be essential to monitor and understand host–pathogen interactions that may contribute to the development of effective vaccines and therapeutics.

## 4. Materials and Methods

### 4.1. Data Collection

Full-length viral nucleotide sequences of 30,983 SARS-CoV-2 genomes were collected from the GISAID EpiCovTM (update: 26 May 2020) [53], belonging to the six geographic areas (according to GISAID database) and distributed in 79 countries as follows: 214 from Africa, 368 from South America, 1590 from Oceania, 2111 from Asia, 6825 from North America, and 19,875 from Europe. The genomes were obtained from samples collected from 24 December 2019 to 13 May 2020.

For each geographical area the collection date of the samples is from 27 February to 1 May for Africa, 24 December to 13 May for Asia, 23 January to 10 May for Europe, 19 January to 12 May for North America, 25 February to 19 April for South America, and 24 January to 21 April for Oceania (Supplementary Table S1).

### 4.2. Variant Calling Analysis

Genome sequences were mapped to the reference sequence Wuhan-Hu-1/2019 (Genbank ID: NC_045512.2) using Minimap v2.12-r847 [54]. The BAM files were sorted by SAMtools sort [55]. The final sorted BAM files were used to call the genetic variants in variant call format (VCF) by SAMtools mpileup and BCFtools [55]. The final call set of the 30,983 genomes was annotated and their impact was predicted using SnpEff v 4.3t [56]. For that, the SnpEff databases were first built locally using annotations of the reference sequence Wuhan-Hu-1/2019 obtained in the GFF format from the NCBI database. Then, the SnpEff database was used to annotate SNPs and InDels with putative functional effects according to the categories defined in the SnpEff manual (http://snpeff.sourceforge.net/SnpEff_manual.html). The frequency of each identified mutation was estimated by normalizing the number of genomes harboring a given mutation, per the total number of genomes recovered from each of the six geographic areas. Non-synonymous mutations with a frequency of 10% or greater were used as a cutoff to define the most frequent mutations [22,57]. Indeed, given that hotspot mutations are known to be strong evidence of positive selection [20] and that sites harboring these mutations have been previously reported under positive selection (http://covid19.datamonkey.org/), we systematically considered the non-synonymous mutation with a frequency >10% in the genomes of six geographical areas as hotspot mutations.

### 4.3. D614G Mutagenesis Analysis

To investigate the possible impact of the most frequent D614G mutation, we conducted an in silico mutagenesis analysis based on the CryoEM structure of the spike protein in its pre-fusion conformation (PDB id 6VSB). Modeling of the D614G mutation was done using UCSF Chimera [58]. Then, the mutant structure was relaxed by 1000 steps of steepest descent (SD) and 1000 steps of conjugate gradient (CG) energy minimizations keeping all atoms with more than 5A from G614 fixed. Comparative analysis of D614 (wild type) and G614 (mutant) interactions with their surrounding residues was done in PyMOL 2.3 (Schrodinger L.L.C).

### 4.4. RBD Mutations and Spike/ACE2 Binding Affinity

Modeling of RBD mutations was performed by UCSF chimera [58] using the 6M0J structure of the SARS-CoV-2 wild-type spike in complex with human ACE2 as a template. Mutant models were relaxed by 1000 steps of SD followed by 1000 steps of CG minimizations keeping all atoms far by more than 5A from the mutated residues fixed. Changes in the binding affinity of the spike/ACE2 complex for each spike mutant were estimated by the MM-GBSA method using the HawkDock server [59].

### 4.5. Clustering and Divergence Analysis

In this work, we use the Jaccard distance to compare the similarity of mutational profile of SARS-CoV-2 genomes between 79 countries. It is a metric particularly suited for clustering and useful when the sets to be compared are of different sizes, because its normalization is designed to take the union of the two sets. We first calculated the mutational frequency in each country individually.

The Jaccard similarity coefficient, also known as the Jaccard index, is defined as the ratio of the size of the intersection (shared mutational profile) divided by the union (union of mutational profiles) of two sets A, B (Equation (1)) [60]:

J (A, B) = (A ∩ B)/(A ∪ B)(1)

Then, the Jaccard index was converted into the Jaccard distance which is noted as the difference between one and the Jaccard similarity coefficient (Equation (2)), and is related to the q-gram distance but without the number of occurrences [61].

d_J_ (A, B) = 1 − J (A, B) (2)

The similarity of the set is based on the Jaccard distance. A distance of zero is equivalent to a 100% overlap between countries.

On the other hand, to calculate the intra-genomic divergence of SARS-CoV-2, we used the Wuhan-Hu-1/2019 genome as a reference sequence, and the other 30,983 genomes were also sorted by country of origin. The divergence was first calculated by estimating the similarities of the genomes with the reference sequence by grouping genomes from the same country using CD-Hit. [62]. All SARS-CoV-2 genomes used in this study were included except those from Ghana which were excluded from this analysis due to the high number of Ns. The percentage of similarity was then recovered to 100%. Then the percentage of divergence for each country was calculated using the following formula (Equation (3)):

(3) $$D=\left(100-\frac{\sum \left(A\times B\right)}{C}\right)$$

A = Similarity percentage; B = Number of genomes with similarity value equal A; C = Total genomes by country ∧ continent; D = Percentage of divergence.

### 4.6. Phylogenetic and Spatio-Dynamic Analysis

We generated a phylogenetic and divergence tree, as well as a genomic epidemiology map based on the 30,983 genomes of SARS-CoV-2 using NextStrain tools (https://nextstrain.org) [63]. The tree was constructed in IQ-TREE v1.5.5 [64] using the maximum likelihood method under the GTR model. The rate of evolution and the time to the most recent common ancestor (TMRCA) were estimated using ML dating in the tree time package [65].

## Figures and Tables

**Table 1:** Impact of mutations on the binding affinity between spike protein RBD and ACE2, evaluated by MM-GBSA binding-free energy calculation (ΔG_Bind_).

Mutations	ΔG_Bind_ (kcal/mol)	ΔΔG ^1^ (kcal/mol)	Effect on Spike/ACE2
V367F	−62.47	3.53	Potentially decreased binding affinity
S477N	−62.69	3.31	
R408I	−62.8	3.2	
V483A	−63.85	2.15	
A522S	−64.03	1.97	
G339D	−64.08	1.92	
N354D	−64.39	1.61	
K356N	−64.81	1.19	
H519Q	−64.84	1.16	
Wild Type	−66	0	Wild-type MMGBSA value
N440K	−67.88	−1.88	Potentially increased binding affinity
N450K	−67.88	−1.88	
D364Y	−68.24	−2.24	
S477R	−69.86	−3.86	

^1^ Binding-free energy change between mutated and wild-type complexes.

**Table 2:** Jaccard distance between countries based on their mutational frequencies. Only the distance less than 0.5 (>50% overlap) between countries is displayed.

Cluster	Sub-Cluster	Countries	Jaccard Distance	Geographic Areas
Cluster 1	SC-1	Brunei, Guam	0.22	Asia, Oceania
Cluster 2	SC-2	Kazakhstan, Georgia	0.22	Asia
Cluster 2	SC-3	Nigeria, Serbia, Croatia, Ireland, Peru	0.26	Africa, Europe, South America
Cluster 2	SC-4	Vietnam, Jordan	0.27	Asia
Cluster 2	SC-5	Sri Lanka, Kuwait	0.30	Asia
Cluster 2	SC-6	Greece, Portugal	0.32	Europe
Cluster 2	SC-7	Singapore, Thailand	0.35	Asia
Cluster 2	SC-8	Finland, Poland	0.35	Europe
Cluster 2	SC-9	Slovenia, Jamaica	0.35	Europe, North America
Cluster 2	SC-10	Denmark, Iceland	0.36	Europe
Cluster 2	SC-11	Germany, Russia	0.36	Europe
Cluster 1	SC-12	Hungary, Latvia	0.41	Europe
Cluster 1	SC-13	Chile, Brazil	0.43	South America
Cluster 1	SC-14	Iran, Pakistan	0.43	Asia
Cluster 2	SC-15	Netherland, Belgium, Austria	0.49	Europe

## Supplementary Materials

The following are available online at https://www.mdpi.com/2076-0817/9/10/829/s1, Figure S1: Dendrogram and heatmap of the hierarchical analysis of clusters between countries. The distance matrix between these countries was calculated using Jaccard distance, and the values ranged from 0 (light yellow) to 1 (red). Table S1: Accession number, collection date, and geographic origins of 30,983 SARS-CoV-2 genomes downloaded from the Gisaid database, Table S2: All genetic variants identified in SARS-CoV-2 genomes from six geographic areas, Table S3: Genetic variants found with a frequency greater than 0.01 in each geographic area, Table S4: Mutations with a non-synonymous effect found in the receptor-binding domain (RBD) of the spike protein, Table S5: Pairwise distances between countries based on their mutation frequencies using the Jaccard index, Table S6: Comparison of siRNA target sequences in the SARS-CoV-2 genome predicted by Chen et al., 2020 with their mutations found in the 30,983 sequences of our study.
