# Supplementary material for: Genomic Diversity and Hotspot Mutations in 30,983 SARS-CoV-2 Genomes: Moving Toward a Universal Vaccine for the “Confined Virus”?
Source: Pathogens. 2020 Oct 10;9(10):829. doi: 10.3390/pathogens9100829 (PMC7600297; doi:10.3390/pathogens9100829)
Supplement: Supplementary file 1 [file pathogens-09-00829-s001.zip › Supplementary Materials/Figure S1.pdf]

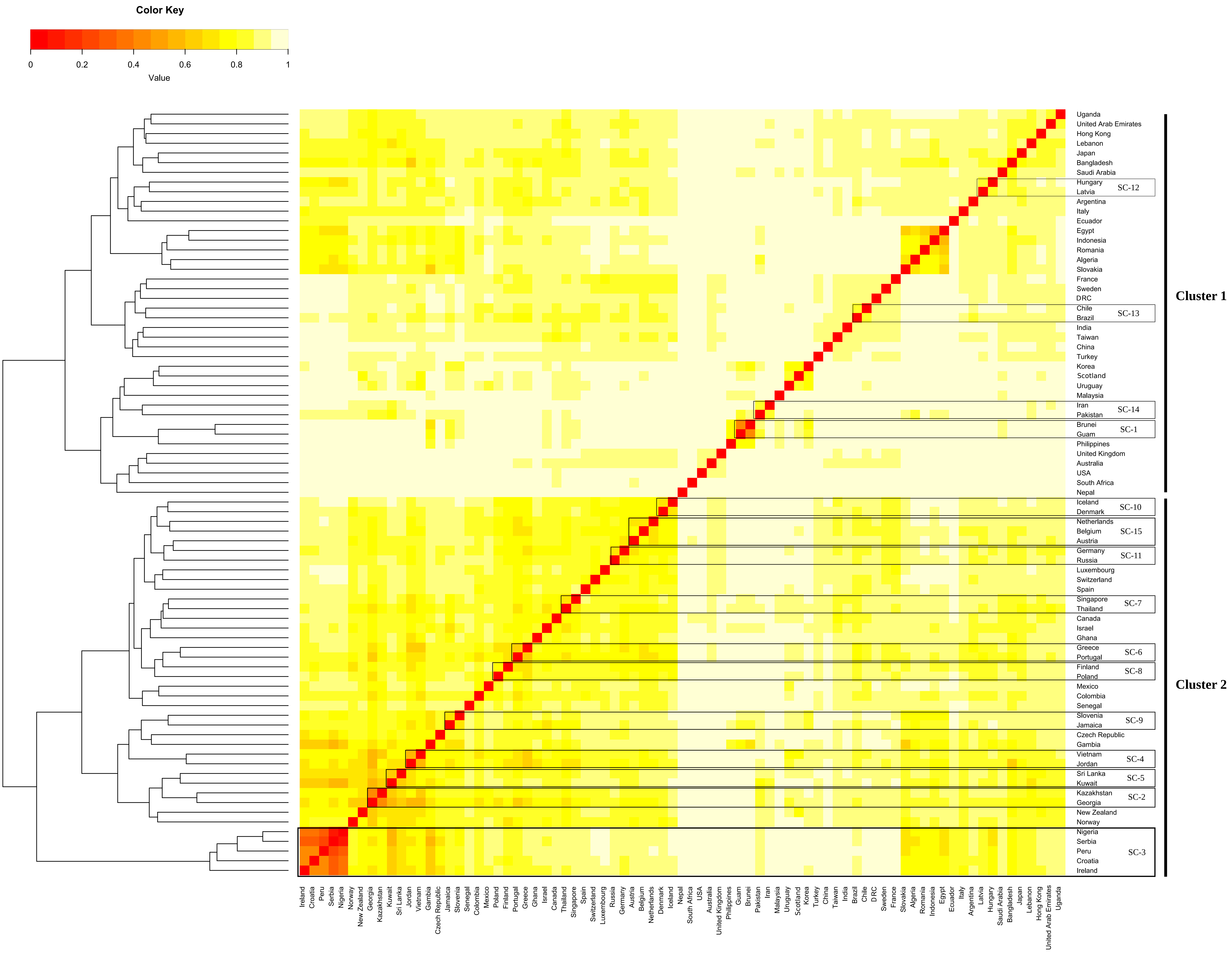

Color Key
0
0.2
0.4
0.6
0.8
1
Value
Uganda
United Arab Emirates
Hong Kong
Lebanon
Japan
Bangladesh
Saudi Arabia
Hungary
Latvia
SC-12
Argentina
Italy
Ecuador
Egypt
Indonesia
Romania
Algeria
Slovakia
France
Sweden
DRC
Chile
Brazil
SC-13
India
Taiwan
China
Turkey
Korea
Scotland
Uruguay
Malaysia
Iran
Pakistan
SC-14
Brunei
Guam
SC-1
Philippines
United Kingdom
Australia
USA
South Africa
Nepal
Iceland
Denmark
SC-10
Netherlands
Belgium
Austria
SC-15
Germany
Russia
SC-11
Luxembourg
Switzerland
Spain
Singapore
Thailand
SC-7
Canada
Israel
Ghana
Greece
Portugal
SC-6
Finland
Poland
SC-8
Mexico
Colombia
Senegal
Slovenia
Jamaica
SC-9
Czech Republic
Gambia
Vietnam
Jordan
SC-4
Sri Lanka
Kuwait
SC-5
Kazakhstan
Georgia
SC-2
New Zealand
Norway
Nigeria
Serbia
Peru
Croatia
Ireland
SC-3
Cluster 1
Cluster 2
Ireland
Croatia
Peru
Serbia
Nigeria
Norway
New Zealand
Georgia
Kazakhstan
Kuwait
Sri Lanka
Jordan
Vietnam
Gambia
Czech Republic
Jamaica
Slovenia
Senegal
Colombia
Mexico
Poland
Finland
Portugal
Greece
Ghana
Israel
Canada
Thailand
Singapore
Spain
Switzerland
Luxembourg
Russia
Germany
Austria
Belgium
Netherlands
Denmark
Iceland
Nepal
South Africa
USA
Australia
United Kingdom
Philippines
Guam
Brunei
Pakistan
Iran
Malaysia
Uruguay
Scotland
Korea
Turkey
China
Taiwan
India
Brazil
Chile
DRC
Sweden
France
Slovakia
Algeria
Romania
Indonesia
Egypt
Ecuador
Italy
Argentina
Latvia
Hungary
Saudi Arabia
Bangladesh
Japan
Lebanon
Hong Kong
United Arab Emirates
Uganda
